# Integrative Literature Review on the Lived Experiences of Parents of Children with a Rare Disease

**DOI:** 10.3390/healthcare14111437

**Published:** 2026-05-22

**Authors:** Assunta Guillari, Keti Ballfusha, Chiara Palazzo, Maurizio Di Martino, Vincenza Giordano

**Affiliations:** 1 Department of Translational Medical Sciences, Clinical Research Center DEMeTra, University of Naples “Federico II”, 80138 Naples, Italy; assunta.guillari@unina.it; 2 Department for Research and Clinical Management of Oncology Care Pathways in the Abdominal District, Istituto Nazionale Tumori-IRCCS-Fondazione G. Pascale, 80138 Naples, Italy; ballfushaketi@gmail.com; 3 Department of Biomedicine and Prevention, University of Rome “Tor Vergata”, 00133 Rome, Italy; 4 Department of Oncology, Hematology and Cellular Therapies, Santobono Pausilipon Hospital, 80122 Naples, Italy; 5 Department of Translational Medical Sciences, University of Naples “Federico II”, 80138 Naples, Italy; maurizio.dimartino@unina.it; 6 Department of Public Health, University of Naples “Federico II”, 80138 Naples, Italy; enza-giordano@hotmail.it

**Keywords:** rare diseases, parents, caregiving burden, diagnostic odyssey, family-centred care, coping, integrative review

## Abstract

Background/Objectives: Rare diseases have a substantial impact not only on affected individuals but also on their families, particularly parents who assume primary caregiving roles. Despite increasing attention to rare conditions, parents’ experiences remain fragmented across the literature. This integrative review aimed to synthesise existing evidence on the experiences and multidimensional impact of caring for a child with a rare disease on parents. Methods: An integrative review was conducted following Whittemore and Knafl’s methodology and reported according to PRISMA 2020 guidelines. A systematic search was performed across MEDLINE, CINAHL, PsycINFO, PsycARTICLES, and Scopus from 1 November 2025 to 31 January 2026. Twenty-two studies (qualitative, quantitative, mixed-methods, and reviews) were included. Data were analysed using thematic synthesis. Results: Three interrelated themes were identified: (1) the diagnostic journey, characterised by prolonged uncertainty, fragmented care, and the pivotal role of communication; (2) multidimensional caregiving burden, encompassing emotional, social, economic, and physical impacts, with notable gender differences; and (3) adaptive trajectories, involving dynamic coping processes, parental upskilling, and meaning-making. Across studies, caregiving burden emerged as a cumulative and system-influenced phenomenon, while adaptation was found to coexist with ongoing uncertainty rather than representing a linear resolution. Conclusions: Caring for a child with a rare disease profoundly affects parents across multiple domains. The findings highlight the need for integrated, family-centred care models, improved diagnostic communication, and sustained psychosocial support. Implications for nursing practice: Nurses play a key role in recognising caregiver burden, supporting adaptive processes, and promoting effective communication throughout the diagnostic and care trajectory.

## 1. Introduction

Rare diseases represent a vast and complex group of conditions that, despite their low individual prevalence, collectively represent a major challenge for healthcare systems, patients, and their families.

Approximately 7000 rare diseases have been identified worldwide [1]. Nguengang Wakap et al. (2020) estimated that rare diseases collectively affect between 3.5% and 5.9% of the global population, corresponding to approximately 263–446 million people worldwide [2]. In the European Union, a disease is defined as rare when it affects fewer than 5 in 10,000 individuals [3]. Using the same prevalence estimate, Nguengang Wakap et al. (2020) estimated that rare diseases affect approximately 17.8–30.3 million people in the European Union [2].

According to the Orphanet database analysis by Nguengang Wakap et al. (2020), 69.9% of rare diseases have an exclusively paediatric onset and 71.9% are genetic in origin [2]. Rare diseases are often severe and life-threatening in childhood; approximately 30% of children affected by a rare disease die before the age of five [4]. These conditions contribute to a severely impaired quality of life [5] and are often chronic, progressive, degenerative, and life-threatening [6]. Despite advances in orphan drug development, only a small minority of rare diseases currently have approved disease-specific medicines [7]. Children with rare diseases often experience multiple disabilities that impair physical, cognitive, behavioural, and social development [8]. The burden of these conditions is not limited to patients: family members, particularly parents, are deeply involved in the day-to-day management of the disease. They take on a central role in ensuring continuous care, coordinating clinical and educational interventions, and acting as medical navigators among different health and social services [8]. This involvement often results in substantial emotional, physical, and financial burden [9].

A particularly critical aspect of the care trajectory is the “diagnostic odyssey”, namely the long and complex path many parents undertake before obtaining a diagnosis for their child. This period is marked by profound uncertainty, frustration, and anxiety, as the child’s symptoms often remain unexplained and the lack of answers fosters feelings of powerlessness and isolation [10,11,12].

For many families, the moment a diagnosis is communicated represents an existential turning point. Receiving a diagnosis—even when painful—brings an end to uncertainty and enables an initial attempt to redefine the parental role within a new framework of meaning [13].

However, how this information is delivered has a decisive impact on parents’ lived experience. When communication is abrupt, impersonal, or lacking in empathy, it can intensify feelings of shock and confusion and leave parents feeling poorly supported. Conversely, sensitive, empathic, and clear communication is associated with higher levels of trust in healthcare professionals and facilitates parents’ processing of the information and navigation of subsequent steps in care [14,15].

In the period immediately following diagnosis, parents report a wide range of emotional responses and practical needs, often characterized by conflicting feelings: relief at finally having a name for their child’s condition, alongside grief, a sense of loss, fear for the future, and guilt [11].

In rare diseases, the day-to-day care pathway is marked by challenges that extend well beyond medical management. Parents often become coordinators of a frequently fragmented system of care [8], acting as intermediaries among schools, hospitals, therapists, and social services [16,17]. In some cases, they give up their careers to provide care for their child [17,18]. This process entails a profound reorganisation of family and couple dynamics, with a substantial impact on psychological, social, and economic well-being [15,19].

Having a child with a rare disease profoundly alters family equilibrium, parental roles, and caregivers’ existential perspectives. Understanding how parents navigate during this turning point, what resources they mobilise, which barriers they encounter, and which forms of support are most effective, is essential for developing family-centred, culturally sensitive, interprofessional models of care that can respond meaningfully to families’ real-world needs. Although the literature on this topic is steadily growing, it remains fragmented and heterogeneous, and it still lacks an integrated perspective capable of capturing the complexity and uniqueness of the parental lived experience in this context.

In light of this evidence, there is a need to systematically investigate the lived experience of parents of children with rare diseases, with particular attention to the critical phases of diagnostic uncertainty and the search for a diagnosis, the process of psychological and relational adaptation in parents’ lives, and the experiences, needs, and coping strategies that emerge throughout the everyday care trajectory.

This integrative review therefore arises from the urgent need to systematise the knowledge currently available, providing a critical and in-depth synthesis of the scientific evidence on the experiences of parents of children with rare diseases. The aim is twofold: first, to address a persisting knowledge gap in the international literature; and second, to generate practical implications to inform healthcare policy, professional training, and the design of effective, sustainable, evidence-based psychosocial interventions.

The aim of this integrative review is to critically synthesise the existing scientific literature on the lived experiences of parents caring for children and adolescents with a rare disease, with particular attention to diagnosis-related experiences, including the diagnostic odyssey and the communication of diagnosis, and to the multidimensional caregiving burden that emerges following diagnosis.

Specifically, the review seeks to examine how diagnostic uncertainty and disclosure processes shape parental emotional, relational, and psychosocial trajectories over time, how these experiences contribute to caregiving burden, and how families subsequently engage in coping and adaptive processes that influence family dynamics and support needs along the care pathway.

The following research questions were formulated to guide the review:

1. How do parents experience the period of uncertainty and diagnostic pursuit (the “diagnostic odyssey”) prior to receiving a rare disease diagnosis for their child?
2. What emotional, cognitive, and relational experiences do parents report at the time the rare disease diagnosis is communicated?
3. What experiences, needs, coping strategies, and adaptive processes emerge in the everyday care trajectory of parents of children and adolescents with a rare disease?
4. How does a rare disease reshape parents’ roles, responsibilities, and day-to-day life balance within their caregiving experience?

## 2. Materials and Methods

### 2.1. Study Design

This integrative review was conducted following the methodological framework proposed by Whittemore and Knafl [20], which enables the inclusion and synthesis of heterogeneous evidence, including qualitative, quantitative, and mixed-methods studies, as well as review articles. This approach was considered particularly appropriate given the complexity of the phenomenon under investigation and the methodological diversity of the available literature.

Unlike traditional systematic reviews focused on homogeneous study designs, the integrative approach allows for a more comprehensive understanding of multifaceted phenomena by combining empirical and theoretical evidence. In the context of rare diseases, where research is often fragmented across disciplines and methodologies, this approach facilitates the development of a more nuanced and holistic interpretation of parents’ lived experiences.

The review was conducted in accordance with the Preferred Reporting Items for Systematic Reviews and Meta-Analyses (PRISMA 2020) guidelines where applicable [21]. The review process followed a structured and iterative approach, including: (1) formulation of the research questions; (2) development of the search strategy; (3) application of inclusion and exclusion criteria; (4) critical appraisal of study quality; (5) data extraction and thematic synthesis; and (6) integrative interpretation of findings.

The review protocol was registered on the Open Science Framework (OSF) on 2 February 2026, before completion of the synthesis and manuscript finalization procedures (registration DOI: 10.17605/OSF.IO/3R7TE).

### 2.2. Search Strategy

The systematic search was conducted across MEDLINE, CINAHL, PsycINFO, PsycARTICLES, and Scopus from 1 November 2025 to 31 January 2026, before manuscript submission. No publication date restrictions were applied. The search strategy was developed through preliminary scoping searches and was structured around three key concepts: (1) rare diseases, (2) parents or caregivers, and (3) psychosocial experiences and impact. Both controlled vocabulary (e.g., Medical Subject Headings [MeSH]) and free-text terms were used to maximise sensitivity. For the condition of interest, the MeSH term “Rare Diseases” and related keywords such as “rare disease”, “rare disorder”, and “orphan disease” were included. For the population, terms included “parent”, “mother”, “father”, “caregiver”, “carer”, “family”, “relative”, and “spouse”. For the outcome domain, terms related to lived experience and psychosocial impact were used, including the MeSH term “quality of life” and keywords such as “psychological burden”, “social burden”, “financial burden”, “emotional burden”, “experiences”, and “diagnostic communication”. Search terms were combined using Boolean operators (AND, OR) and adapted to the specific requirements of each database. Where available, searches were conducted within the title, abstract, and subject heading fields. Preliminary versions of the search strings were tested and refined to optimise sensitivity while minimising irrelevant results.

To enhance reproducibility, the complete PubMed/MEDLINE search string is reported here as an example:(“Rare Diseases”[MeSH Terms] OR “orphan disease”[TIAB] OR “rare disorder”[TIAB] OR “rare disease”[TIAB]) AND (“caregiver”[TIAB] OR “family”[TIAB] OR “carer”[TIAB] OR “parent”[TIAB] OR “mother”[TIAB] OR “father”[TIAB] OR “relative”[TIAB] OR “spouse”[TIAB]) AND (“quality of life”[MeSH Terms] OR “quality of life”[TIAB] OR “experiences”[TIAB] OR “diagnosis communication”[TIAB] OR “psychological burden”[TIAB] OR “social burden”[TIAB] OR “financial burden”[TIAB] OR “emotional burden”[TIAB]). All searches were conducted independently by two reviewers, and results were managed using Rayyan, Intelligent Systematic Review software to facilitate screening and reduce the risk of bias [22]. Any disagreements were resolved through discussion and consensus between reviewers. The full search strategies for each database, including all keywords, combinations, and search fields, together with the number of results obtained for each strategy, are reported in Supplementary Materials Table S1.

### 2.3. Inclusion and Exclusion Criteria

Eligibility criteria were defined in alignment with the research questions and the aims of the review.

Inclusion criteria

Studies were included if they met the following criteria:

- Population: Parents (mothers and/or fathers) or informal family caregivers (non-professional) of children or adolescents (<18 years) diagnosed with a rare disease.
- Condition of interest: Rare diseases as defined by international or national regulatory frameworks (e.g., European Union definition: prevalence < 5 per 10,000), or conditions explicitly described as rare by the study authors.
- Phenomenon of interest: Studies addressing at least one of the following domains related to parental experience:
  ○ Lived experiences and caregiving perceptions;
  ○ Emotional, psychological, social, physical, or economic burden;
  ○ Quality of life and psychosocial outcomes;
  ○ Experiences related to the diagnostic process, including diagnostic uncertainty, diagnostic odyssey, and/or communication of diagnosis.
- Study design: Empirical studies using qualitative, quantitative, or mixed-methods designs, as well as secondary research (e.g., systematic reviews, scoping reviews, rapid reviews, and meta-syntheses), in accordance with the integrative review methodology.
- Language: Articles published in English or Italian.
- Publication type: Peer-reviewed journal articles.

Exclusion criteria

Studies were excluded if they met any of the following criteria:

- Population mismatch: Studies focusing exclusively on adult patients, professional caregivers or healthcare providers without parental perspectives, or other family members without disaggregated data on parents.
- Condition mismatch: Studies addressing conditions not classified or described as rare diseases.
- Outcome mismatch: Studies not reporting parental experiences, caregiving impact, psychosocial outcomes, or diagnosis-related experiences.
- Study type: Editorials, commentaries, opinion papers, conference abstracts, dissertations, grey literature, or methodological papers without relevant empirical data.
- Language: Studies published in languages other than English or Italian.

Articles published in English or Italian were included. This criterion referred to the language of publication and not to the country in which the study was conducted. The language restriction was applied for feasibility reasons, as these were the languages in which the review team could ensure full-text assessment, data extraction, and interpretation without translation-related loss of meaning. In line with the integrative review approach, both primary and secondary studies were considered eligible. However, review articles were not treated as primary data sources for theme generation. To minimize the risk of double counting, the primary studies included in the review articles were compared with the primary studies included in the present review. When overlap was identified, empirical findings were extracted and synthesised from the primary study rather than counted again through the review article. Review articles were used only for contextual triangulation, comparison with broader evidence, and interpretation of the consistency of the identified patterns across the wider literature. Grey literature, dissertations, conference abstracts, preprints, and non-peer-reviewed sources wereexcluded, as this review focused on evidence published in peer-reviewed journals to enhance the methodologicalconsistency, reliability, and comparability of the included studies.

### 2.4. Study Selection and Data Management

The literature search identified a total of 2391 records: 391 from MEDLINE, 691 from CINAHL, 305 from PsycInfo and PsycArticles, and 1004 from Scopus. After removal of duplicates, 2054 records remained for title and abstract screening.

**Figure 1:**
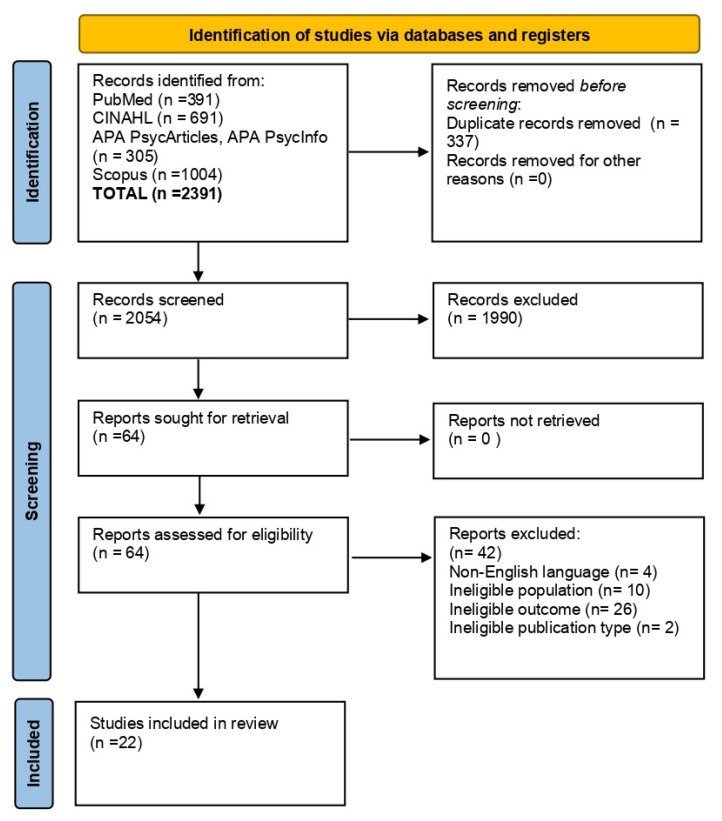
PRISMA 2020 flow diagram of the study selection process [21].

Two independent reviewers screened titles and abstracts, resulting in 64 potentially eligible studies for full-text assessment. Of these, 42 were excluded for not meeting the inclusion criteria. A total of 22 studies were included in the final synthesis. The entire process of identification, screening, eligibility, and inclusion was documented and represented graphically according to the PRISMA 2020 flowchart (Figure 1), adapted to the methodological context of integrative review, following the PRISMA guidelines [21].

### 2.5. Critical Appraisal of Study Quality

The methodological quality and risk of bias of the included studies were assessed in accordance with the principles of integrative reviews, which emphasise methodological transparency while accommodating heterogeneous evidence [20,23].

Given the diversity of study designs, appraisal tools appropriate to each methodology were applied. The QUADS tool (Quality Appraisal for Diverse Studies) was used to assess qualitative, mixed-methods, and review studies [24], while the Joanna Briggs Institute (JBI) Critical Appraisal Checklist was used for quantitative cross-sectional studies [25].

For the JBI checklist, scores ranged from 0 to 8 and were classified as high (7–8), moderate (5–6), or low (<5). For the QuADS tool, the maximum score was 39, with thresholds of high (30–39), moderate (20–29), and low quality (<20).

All appraisals were conducted independently by two reviewers, and disagreements were resolved through discussion and consensus. Quality appraisal informed the interpretation of findings but was not used to exclude studies or to apply formal weighting. Instead, studies were considered within the synthesis with attention to their methodological strengths and limitations.

The methodological appraisal indicated an overall satisfactory quality of the included studies. Most qualitative studies [10,26,27,28,29,30,31,32,33], both mixed-methods studies [34,35], all review studies [9,18,19,36] and two quantitative studies [37,38] were rated as high quality, whereas four quantitative studies [4,39,40,41] and one qualitative study [42] were rated as moderate quality; no study was rated as low quality.

Across studies assessed with QuADS, the lowest-scoring domain was stakeholder involvement in the design or conduct of the research. In qualitative, mixed-methods, and review studies, additional weaker areas included theoretical underpinning, sampling adequacy, justification of data collection methods, and transparency in reporting methodological limitations. In the quantitative studies assessed with the JBI checklist, the main limitations concerned the identification and management of confounding factors, with some uncertainty also noted regarding the use of objective and standard criteria for measuring the condition.

These methodological considerations were taken into account when interpreting the findings and in assessing the overall confidence in the evidence. The results of the quality assessment are reported in Supplementary Materials Table S2 (QUADS) and Table S3 (JBI).

### 2.6. Data Extraction and Synthesis

A total of 22 studies were included, comprising 6 quantitative studies, 10 qualitative studies, 2 mixed-methods studies, and 4 review studies. Data were extracted from the included studies using ad hoc extraction charts developed for this review. The extraction charts were designed to capture the following information: authors and year of publication, country, title, study aim, study design, sample and setting, data collection tools or methods, main findings, declared limitations, and quality rating. Detailed data extraction for the included studies is reported in the Supplementary Materials (Table S4 for quantitative studies, Table S5 for mixed-methods studies, Table S6 for qualitative studies and Table S7 for review studies).

Data extraction and synthesis followed the integrative review process described by Whittemore and Knafl [20], involving data reduction, comparison, and thematic categorisation of findings across studies.

Extracted data included study characteristics (e.g., design, sample, setting), key findings, and methodological quality. Data were systematically compared and grouped to identify recurring patterns, similarities, and differences across studies.

Findings were synthesised using a narrative and thematic approach, allowing integration of evidence from heterogeneous study designs. This process enabled the identification of overarching themes that capture the complexity of parents’ experiences in the context of rare diseases.

Given the heterogeneity of study designs, populations, measures, and outcomes, findings were not statistically pooled. Instead, synthesis was conducted using a convergent integrative approach. Qualitative findings were treated as experiential and interpretative evidence, whereas quantitative findings were used to identify the direction, magnitude, and recurrence of burden-related outcomes, such as stress, quality of life, healthcare satisfaction, and perceived caregiving burden. Mixed-methods studies were examined by integrating their qualitative and quantitative components according to the phenomenon addressed. Review articles were not combined with primary study data in the thematic synthesis. Rather, they were used for contextual triangulation and to assess whether the patterns identified in primary studies were consistent with broader evidence. During synthesis, greater interpretative weight was given to findings that showed convergence across study designs or that were supported by higher-quality studies. Divergent or design-specific findings were retained and discussed where relevant.

Accordingly, the three themes were generated primarily from the primary qualitative, quantitative, and mixed-methods studies, whereas review articles contributed to contextual interpretation and triangulation but did not independently determine theme development.

## 3. Results

### 3.1. Characteristics of Included Studies

The 22 included studies were published between 2015 and 2026. Ten studies adopted qualitative designs [10,26,27,28,29,30,31,32,33,42], six were quantitative [4,37,38,39,40,41], two used mixed-methods approaches [34,35], and four were review studies [9,18,19,36].

Most studies involved parents of children with rare diseases, although two qualitative studies included mothers only [26,28], and one quantitative study focused on female caregivers only [39].

The research was conducted in a variety of geographical settings. The qualitative studies were conducted across the USA, Australia, Turkey, Ireland, and Germany; the quantitative studies across the USA, Croatia, Ireland, Poland, and Australia, with one international European study; the mixed-methods studies in Taiwan and one study with no country reported; whereas all review studies were international in scope (Tables S4–S7).

### 3.2. Themes Emerging from the Included Studies

The analysis of the included studies identified three interrelated themes reflecting a dynamic trajectory in parents’ experiences of caring for a child with a rare disease (Table 1). The key sources reported in Table 1 correspond to studies included in the review and were selected to illustrate the most representative evidence supporting each theme. Secondary studies were used for contextual triangulation.

The first theme, the diagnostic journey, encompassed experiences of prolonged uncertainty and diagnostic odyssey, perceived fragmentation of care and lack of recognition of parental knowledge, the emotional complexity of diagnostic disclosure, and the pivotal role of communication quality.

The second theme, multidimensional caregiving burden, captured the cumulative impact of caregiving across emotional, relational, social, economic, and physical domains, including gendered patterns of caregiving, family reorganisation, social isolation, and financial strain.

The third theme, adaptive trajectories and family reconfiguration, described the dynamic processes through which parents develop coping strategies, acquire new competencies, and engage in meaning-making and identity transformation over time.

Together, these themes illustrate a process that unfolds across the care trajectory, moving from diagnostic uncertainty to cumulative burden and subsequent adaptive reconfiguration.

To enhance transparency in the integration of heterogeneous evidence, each theme was mapped according to the type of evidence supporting it and the degree of convergence across studies. Themes supported by both qualitative and quantitative evidence were interpreted as showing stronger convergence, whereas themes primarily supported by qualitative or review evidence were interpreted more cautiously as contextually grounded patterns.

Main theme:

1. The Diagnostic Journey: from uncertainty to disclosure and validation

Across the included studies, the diagnostic journey emerged as a central phase in parents’ experiences, characterised by prolonged uncertainty, fragmented care pathways, and complex interactions with healthcare professionals. This trajectory, commonly described as the “diagnostic odyssey”, involved significant emotional and relational challenges for families.

  1.1. Prolonged uncertainty and diagnostic odyssey.
    Parents consistently reported a prolonged and often demanding search for answers prior to receiving a definitive diagnosis. This phase was characterised by repeated consultations, multiple investigations, evolving clinical hypotheses, and, in some cases, misdiagnoses. Quantitative evidence indicated that many families consulted several specialists before receiving a diagnosis and perceived that an earlier diagnosis might have been possible [41]. Qualitative findings described persistent uncertainty, anxiety, and anticipatory fear during this period [10,28]. The absence of a diagnostic label was associated with difficulties in accessing healthcare services, educational support, and social recognition of the child’s condition. For some families, particularly those with older children or adolescents, prolonged diagnostic processes were also associated with tensions related to the continuation of diagnostic investigations and decision-making about further testing [30].
    Where reported, the duration of the diagnostic odyssey varied substantially across studies. In paediatric-focused samples, reported durations ranged from less than 1 year to more than 5 years, with some studies reporting delays of 13–15.6 years among diagnosed or still undiagnosed children [34,41]. Deuitch et al. reported that the time from symptom onset to diagnosis ranged from 1.5 to 13 years among diagnosed children and from 2 to 14 years among children who remained undiagnosed [29]. In one study focusing on emerging-ultrarare disorders, the median length of the diagnostic search was 9.8 years, with a reported range of 1.11–48.82 years [32]. Because only a subset of included studies reported comparable numerical estimates, and because some studies included ongoing undiagnosed cases, no pooled average was calculated.
  1.2. Epistemic injustice and fragmentation of care.
    Several studies highlighted that parents’ experiences during the diagnostic process were shaped not only by clinical complexity but also by interactions with healthcare professionals. Parents frequently reported feeling unheard or not taken seriously, with their knowledge of their child sometimes being overlooked or minimised [31]. These experiences were associated with increased emotional distress and reduced trust in healthcare providers. In response, many parents described assuming active roles in coordinating care, organising appointments, and monitoring clinical information. Limited continuity of care, combined with the involvement of multiple specialists, further contributed to fragmentation and increased caregiving demands. Parents also engaged in independent information-seeking, including the use of online resources and peer support networks, to better understand their child’s condition and inform decision-making [29]. While these strategies provided support, they also reflected gaps in formal healthcare communication and coordination.
  1.3. The moment of disclosure: shock, validation, and ambivalence.
    Receiving the diagnosis represented a critical turning point in the parental trajectory. Across studies, this moment was characterised by a complex interplay of emotional responses, including shock, grief, anger, devastation, and, in some cases, relief [27,28,34]. Relief was typically associated with the end of uncertainty and the validation of longstanding parental concerns. For many families, the diagnostic label enabled access to services, connection with support networks, and the possibility of future planning. However, the diagnosis also marked the beginning of a new phase of uncertainty, particularly in ultra-rare or genomically emerging conditions, where limited prognostic information was available [32]. Thus, diagnosis did not always equate to closure; rather, it often signified a transition from uncertainty about “what” to uncertainty about “what next.”
  1.4. Communication quality as a turning point.
    The quality of communication at the time of diagnosis emerged as a key factor influencing parents’ experiences. Parents valued clear, empathic, and responsive communication, as well as opportunities to ask questions and receive ongoing support [27,31]. Conversely, insufficient, delayed, or poorly structured communication was associated with increased distress and dissatisfaction. The communication process influenced both immediate emotional responses and subsequent engagement with healthcare services, including trust in professionals and adherence to care pathways.

2. The Multidimensional Caregiving Burden

Across the included studies, caregiving burden was described as a multidimensional phenomenon involving emotional, relational, social, economic, and physical domains. These dimensions frequently co-occurred and interacted over time, contributing to sustained pressure on families.

  2.1. Emotional strain as a pervasive and gendered experience.
    Emotional burden was consistently reported across studies, with parents describing elevated levels of stress, anxiety, depressive symptoms, and ongoing uncertainty [37,38,39]. These outcomes were associated with diagnostic ambiguity, caregiving demands, and perceived lack of support from healthcare systems. Gender differences were evident, with mothers more frequently identified as primary caregivers and reporting higher levels of stress and reduced quality of life compared to fathers [26,35]. Qualitative findings suggested that fathers also experienced distress, although it was often expressed through different coping patterns and roles within the family.
  2.2. Family system reconfiguration and relational strain.
    Caregiving was associated with substantial changes in family roles and routines. Parents reported reduced time for couple relationships, increased conflict, and challenges in maintaining family balance [26,33]. Caregiving responsibilities often extended beyond direct care to include coordination of services, administrative tasks, and advocacy, contributing to role overload. Quantitative studies also reported associations between caregiving burden and reduced health-related quality of life across multiple domains [36,38].
  2.3. Social isolation, stigma, and reduced participation.
    Social impact emerged as a recurring theme, with parents reporting reduced participation in social activities, limited peer interactions, and feelings of isolation [9,18].
    Limited public awareness of rare diseases contributed to experiences of stigma and the need to repeatedly explain the child’s condition. These experiences were associated with social withdrawal and reduced engagement in community and leisure activities.
  2.4. Economic and physical consequences of sustained caregiving.
    Economic burden included both direct and indirect costs, such as treatment expenses, travel, specialised care, and reduced employment, particularly among mothers [38,40].
    Physical effects, including fatigue, sleep disturbances, and somatic symptoms, were also reported [9], highlighting the broader impact of sustained caregiving demands on parental health and daily functioning.
    Across studies, the systems most frequently implicated in the production or amplification of caregiving burden were healthcare services, educational services, and social welfare systems. Healthcare systems contributed through fragmented care pathways, repeated consultations, poor coordination, and limited recognition of parental expertise. Educational and social systems contributed to difficulties in accessing appropriate school support, disability-related resources, respite care, and welfare benefits. Employment-related systems were also implicated when parents, particularly mothers, reduced working hours or left employment to meet caregiving demands.

3. Adaptive Trajectories and Family Reconfiguration.

In addition to challenges, studies described adaptive processes through which parents adjusted to caregiving over time. These processes were shaped by individual, relational, and contextual factors.

  3.1. Coping as a dynamic and relational process.
    Parents reported a range of coping strategies, including problem-focused approaches (e.g., information-seeking, care coordination, and advocacy) and emotion-focused strategies (e.g., acceptance, cognitive reframing, and social support) [9,37].
    Coping strategies varied depending on disease characteristics, available support, and family dynamics. Gender differences in coping patterns were also described, reflecting differentiated roles within caregiving [35]. Greater access to social support and positive healthcare experiences were associated with lower levels of psychological distress [39].
  3.2. Parental upskilling and identity transformation.
    Several studies described the progressive acquisition of knowledge and skills by parents, enabling them to actively participate in care and decision-making [9,18].
    Parents frequently developed competencies in navigating healthcare systems, interpreting medical information, and coordinating services. In some cases, particularly in ultra-rare conditions, parents assumed roles as advocates or key coordinators of care [32].
  3.3. Meaning-making and rebalancing family priorities.
    Qualitative findings highlighted processes of meaning-making and adjustment of expectations over time [9,42]. Parents described shifts in priorities, increased attention to daily achievements, and ongoing negotiation of future expectations.
    Adaptation often involved adjustments in response to changes in the child’s condition or developmental stage, requiring continuous re-evaluation of goals and caregiving strategies [30].

Final synthesis of themes

Together, these findings describe a trajectory in which parents move from diagnostic uncertainty to ongoing caregiving challenges and subsequent adaptive processes. The themes illustrate how experiences evolve over time and are shaped by interactions between individual, relational, and healthcare system factors.

## 4. Discussion

This integrative review provides a comprehensive synthesis of the lived experiences of parents of children with rare diseases, highlighting a dynamic trajectory that unfolds from diagnostic uncertainty to cumulative caregiving burden and subsequent adaptive reconfiguration. By integrating qualitative, quantitative, and mixed-methods evidence, the findings move beyond fragmented accounts of parental experience and suggest that these dimensions are interconnected across the care pathway rather than occurring as isolated phenomena [9,18]. While previous studies have extensively documented the impact of rare diseases on families, they have often examined diagnostic experiences, caregiving burden, and coping processes separately. The present synthesis suggests that these elements are better understood as part of a continuous and evolving process shaped by both individual and systemic factors. This interpretation is consistent with broader epidemiological and health systems evidence highlighting the long-term and multidimensional impact of rare diseases on patients and families [2].

The main conceptual contribution of this review is the proposal of a trajectory-based conceptual framework of parental experience in paediatric rare diseases. This framework does not present the identified themes as fixed or linear stages, but as interconnected and recursive dimensions of parental experience. Diagnostic uncertainty and disclosure shape the way parents enter the care trajectory; cumulative caregiving burden develops across emotional, relational, social, economic, and physical domains; and adaptive reconfiguration emerges through coping, parental upskilling, meaning-making, and renegotiation of family roles. Importantly, adaptation does not represent the resolution of burden. Rather, it coexists with ongoing uncertainty, fragmented care pathways, and changing needs across the child’s developmental and clinical trajectory.

The diagnostic journey as a critical and relational phase

The diagnostic journey emerged as a central and emotionally charged phase, confirming the “diagnostic odyssey” as a key feature of parental experience [10,28,41]. Beyond prolonged uncertainty, this phase was characterised by complex interactions with healthcare systems, including fragmented care pathways, repeated consultations, and perceived delays in diagnosis. Importantly, the findings suggest that diagnostic uncertainty is not solely attributable to disease complexity, but may also reflect communication gaps, limited continuity of care, and insufficient recognition of parents’ experiential knowledge [29,31]. Parents frequently described feeling unheard or inadequately supported, which contributed to distress and reduced trust in healthcare professionals. The moment of diagnosis represented a turning point, marked by a coexistence of relief and emotional distress. While receiving a diagnosis often validated parental concerns and facilitated access to services, it also introduced new uncertainties, particularly in the context of ultra-rare or poorly understood conditions [27,32]. Crucially, the quality of diagnostic communication emerged as a key factor shaping parents’ subsequent experiences. Consistent with previous research, empathic, clear, and family-centred communication was associated with better emotional adjustment and trust, whereas abrupt or insufficient communication intensified distress and dissatisfaction [27,31]. Taken together, these findings indicate that the diagnostic journey should not be understood solely as a function of clinical uncertainty, but as a relational and system-mediated process in which communication practices, recognition of parental knowledge, and continuity of care play a central role. In this perspective, diagnostic delay may reflect not only biomedical complexity but also structural and interactional limitations within healthcare systems.

Caregiving burden as a multidimensional and cumulative process

Consistent with existing literature, caregiving burden was identified as a multidimensional construct encompassing emotional, relational, social, economic, and physical domains [19,36,38]. However, this review extends previous knowledge by highlighting the cumulative and interrelated nature of these dimensions. Emotional distress, family reorganisation, social isolation, and financial strain were not experienced as separate challenges, but as mutually reinforcing pressures that evolved over time. Emotional burden, including stress, anxiety, and depressive symptoms, was consistently reported across studies and was closely linked to uncertainty, lack of support, and dissatisfaction with healthcare services [37,38,39]. Caregiving also had a profound impact on family dynamics, often requiring a reorganisation of roles and responsibilities, with consequences for couple relationships and broader family functioning [26,33]. At the same time, parents reported significant social isolation and stigma, often related to limited public awareness of rare diseases and the practical constraints associated with caregiving [9,18]. Economic burden also emerged as a critical issue, including both direct costs of care and indirect costs such as reduced employment or job loss, particularly among mothers [38,40]. Taken together, these findings support the interpretation of caregiving burden not as an individual psychological outcome, but as a systemic phenomenon shaped by the interaction between disease complexity, healthcare organisation, and social context. While caregiving burden has traditionally been conceptualised as an individual or family-level outcome, the present synthesis suggests that it is more appropriately understood as a system-generated phenomenon. The interaction between fragmented care pathways, limited access to coordinated support, and sociocultural expectations of caregiving appears to amplify and sustain this burden over time. In this perspective, caregiving burden appears to be co-constructed through the interaction between disease-related demands and systemic limitations, including fragmented care pathways, limited access to coordinated support, and sociocultural expectations surrounding caregiving roles. This suggests that burden should not be interpreted exclusively as an individual or family-level outcome, but as a phenomenon shaped by the organisation and responsiveness of healthcare systems.

Gendered dimensions of caregiving and underrepresentation of fathers

A consistent finding across studies was the presence of a marked gender imbalance in caregiving roles. Mothers were more frequently identified as primary caregivers and, in studies including both mothers and fathers, reported higher levels of stress, lower quality of life, and greater caregiving responsibilities compared to fathers [26,35]. However, these findings should be interpreted cautiously, as several included studies relied on predominantly female or mother-only samples, which may have amplified the apparent predominance of maternal burden. Accordingly, these gender-related findings should be interpreted as indicative of patterns in the available literature rather than as definitive evidence of differences between mothers and fathers. This pattern appears to reflect broader sociocultural and organisational dynamics rather than differences in emotional involvement. While fathers were often less involved in daily caregiving tasks, qualitative evidence suggests that they experienced distress through different coping mechanisms, including emotional internalisation and role redefinition [11,43]. Importantly, the literature remains heavily skewed toward mothers’ perspectives, with fathers underrepresented across studies. This imbalance limits the comprehensiveness of current knowledge and highlights the need for more inclusive research designs that capture the diversity of parental experiences.

Adaptive trajectories: coping, identity transformation, and meaning-making

In addition to burden and disruption, the review identified dynamic adaptive processes through which parents reconfigure roles, identities, and expectations over time. Adaptation did not imply the absence of distress but rather reflected an ongoing negotiation between vulnerability and agency. Parents reported a range of coping strategies, including problem-focused approaches such as information-seeking and care coordination, as well as emotion-focused strategies such as acceptance and cognitive reframing [9,37]. A key aspect of adaptation was the process of “parental upskilling”, whereby parents progressively acquired specialised knowledge and became active participants in care coordination and decision-making [18,32]. This process highlights a shift in the traditional boundaries between families and healthcare systems, with parents increasingly assuming roles that extend beyond caregiving into areas of coordination, knowledge production, and decision-making. While this may enhance parental agency, it also reflects unmet needs within formal care structures, as parents often take on responsibilities that would ideally be supported by coordinated services. Meaning-making processes also emerged as central to adaptation, with parents describing shifts in priorities, expectations, and perceptions of quality of life [30,42]. Taken together, these findings indicate that adaptation should not be understood as a linear or final stage. Rather, coping and adjustment occur alongside ongoing uncertainty and burden, reflecting a continuous process of negotiation between constraint and agency within the context of long-term caregiving.

Gaps in the literature and methodological considerations

Despite growing attention to this topic, several important gaps remain in the literature. First, many studies are based on small, non-probability samples, often recruited through patient organisations, which may limit generalisability [10,26]. Second, the predominance of qualitative designs, while valuable for capturing lived experiences, highlights the need for more longitudinal and mixed-methods research to better understand how parental experiences evolve over time [39]. Third, as noted, fathers remain underrepresented, contributing to a gendered narrative that does not fully reflect the diversity of caregiving experiences. Finally, limited attention has been paid to evaluating structured psychosocial interventions, despite clear evidence of the substantial emotional and social burden experienced by families. Existing studies suggest that peer support and informal networks play a crucial role, but more systematic and evidence-based interventions are needed [15,18].

Implications for practice and healthcare systems

The findings of this review have important implications for clinical practice and healthcare organisation. First, they underscore the need for family-centred models of care that recognise parents as active partners in the care process rather than passive recipients of information. Second, improving the quality of diagnostic communication should be considered a priority, as it has a lasting impact on parents’ emotional well-being and trust in healthcare professionals. Third, greater coordination across healthcare, educational, and social services is essential to reduce fragmentation and alleviate caregiving burden. Finally, the development of structured psychosocial support interventions, including peer support programmes and caregiver-focused services, is crucial to address the multidimensional needs of families and to promote long-term well-being.

To strengthen the practical contribution of this review, the findings may be translated into a family-centred rare disease support pathway. First, caregiver needs should be assessed from the phase of diagnostic suspicion, rather than only after diagnosis. Second, diagnostic disclosure should follow a structured communication protocol that includes preparation of the family, clear and non-technical language, emotional containment, written information, and planned follow-up. Third, a nurse care coordinator or family navigator should be identified to support parents in accessing services, coordinating appointments, and reducing fragmentation across healthcare, educational, and social systems. Fourth, caregiver burden should be routinely screened using brief validated tools or structured clinical assessment, with particular attention to emotional distress, social isolation, fatigue, employment disruption, and financial strain. Fifth, families should be systematically connected with peer support networks, patient organisations, psychological counselling, and social welfare resources. These actions move the findings beyond description and provide a pragmatic framework for implementing family-centred care in rare disease pathways.

Final synthesis

Overall, this review suggests that parental experiences in the context of rare diseases are best understood as a dynamic and evolving process shaped by the interplay between diagnostic uncertainty, cumulative burden, and adaptive reconfiguration. These findings highlight the need for integrated, coordinated, and family-centred approaches that address not only the medical aspects of rare diseases but also their profound psychosocial impact on families. Taken together, these findings suggest that parents’ experiences in the context of rare diseases are shaped not only by the clinical condition, but by how healthcare systems recognise, support, and engage families over time.

## 5. Review Limitations

This integrative review has several methodological limitations that should be considered when interpreting the findings. First, the inclusion of studies published only in English or Italian may have introduced language bias and limited the representation of evidence from non-English- and non-Italian-speaking contexts, where healthcare systems, cultural expectations of caregiving, and family support structures may differ substantially. Therefore, the findings should not be interpreted as fully representative of all sociocultural and healthcare contexts, particularly those in which relevant evidence may be published in other languages. Second, the body of evidence was largely dominated by qualitative studies based on small, non-probability and often culturally homogeneous samples. While this allowed for an in-depth exploration of lived experiences, it may limit the transferability of the findings and may have privileged subjective accounts over more generalisable patterns. A further limitation concerns the marked gender imbalance across the included studies. Several samples were predominantly female, and some studies included only mothers or female caregivers. This sampling bias may have contributed to an overemphasis on maternal caregiving burden and limits the extent to which gender-related findings can be interpreted as reflecting true differences between mothers and fathers. Therefore, findings suggesting higher burden among mothers should be interpreted with caution and understood in light of the underrepresentation of fathers in the available literature. In addition, the exclusion of grey literature, preprints, dissertations, and conference abstracts may have led to the omission of emerging, unpublished, or non-peer-reviewed evidence. However, this decision was made to ensure that the synthesis was based on peer-reviewed studies and to preserve methodological consistency across the included sources. The absence of longitudinal studies restricts understanding of how parental experiences, burden, and adaptive processes evolve over time, particularly across key developmental transitions. Finally, the review did not include a formal assessment of publication bias or sensitivity analyses, which may affect the overall robustness of the synthesis. Despite these limitations, the integrative approach enabled the inclusion of heterogeneous evidence and the triangulation of findings across study designs, strengthening the interpretative depth and internal coherence of the synthesis. However, the results should be interpreted as contextually grounded rather than universally generalisable.

## 6. Conclusions

This integrative review demonstrates that caring for a child with a rare disease is a dynamic and multidimensional process that profoundly affects parents’ psychological, social, and economic well-being. Rather than representing isolated challenges, diagnostic uncertainty, caregiving burden, and adaptive processes appear to be interconnected across the care trajectory. The findings highlight that parental vulnerability is not solely determined by disease severity but is strongly shaped by systemic factors, including fragmented care pathways, inadequate communication, and limited access to coordinated support. Importantly, adaptation should not be interpreted as a resolution of burden but as an ongoing process that coexists with persistent uncertainty and structural challenges. Overall, these results underscore the need to move beyond child-centred models of care towards integrated, family-centred approaches that recognise parents as active partners and address the broader psychosocial impact of rare diseases.

The main contribution of this review is the identification of parental experience as a trajectory shaped by three interconnected processes: diagnostic uncertainty and disclosure, cumulative caregiving burden, and adaptive reconfiguration. These processes should not be addressed separately but through structured, family-centred, and nurse-coordinated support pathways that accompany parents from diagnostic suspicion to long-term care.

## 7. Implications for Nursing Practice

Nurses play a central role in supporting families of children with rare diseases and should adopt a proactive, family-centred approach throughout the care pathway. This includes systematically recognising parents as partners in care and actively involving them in decision-making processes.

Given their continuous contact with families, nurses are well positioned to identify early signs of psychological and social distress, including anxiety, depressive symptoms, isolation, and caregiver fatigue. Routine screening and ongoing assessment of caregiver well-being should therefore be integrated into clinical practice. Nurses should also act as facilitators of caregiver empowerment by providing tailored information, enhancing health literacy, and supporting parents in navigating complex healthcare systems. In this context, strengthening parents’ self-efficacy may mitigate feelings of helplessness and improve coping capacity. Particular attention should be given to critical moments such as diagnostic disclosure. Nurses should promote clear, empathic, and structured communication, ensuring that parents are given adequate time, understandable information, and opportunities to ask questions. Finally, nurses should contribute to improving care coordination by acting as a link between healthcare, educational, and social services, thereby reducing fragmentation and alleviating caregiving burden. From a nursing perspective, these findings support the implementation of a structured family-centred pathway for rare diseases. Nurses should contribute to early caregiver needs assessment, facilitate diagnostic communication, monitor caregiver burden over time, and act as care coordinators across services. In clinical practice, this may include the use of structured checklists during diagnostic disclosure, routine screening for caregiver fatigue and psychosocial distress, referral to psychological and social support services, and systematic connection with patient organisations and peer support networks.

Regarding diagnostic communication, nurses may support the initial disclosure process when organisationally and clinically appropriate, by helping to create a family-centred environment, assessing parents’ immediate emotional responses, clarifying information, and ensuring that follow-up support is planned. However, their most sustained contribution is likely to occur in the post-diagnosis sense-making phase, when parents need help interpreting information, identifying care priorities, navigating services, monitoring caregiver burden, and connecting with psychosocial and peer support resources.

From a nursing perspective, the adaptive trajectories identified in this review can be translated into two concrete interventions: a nurse-led case management role and structured peer support integration. A nurse care coordinator or family navigator could support families from the phase of diagnostic suspicion by assessing caregiver needs, facilitating communication after diagnostic disclosure, coordinating appointments across specialist, primary care, educational, and social services, monitoring caregiver burden over time, and ensuring referral to psychological, social welfare, and patient-organisation resources. In parallel, structured peer support integration should connect parents with patient organisations, disease-specific or symptom-based support groups, and families with similar lived experiences, while helping them evaluate the reliability and emotional impact of information obtained through peer networks.

## 8. Implications for Future Research

Future research should prioritise longitudinal study designs to better understand how parental experiences, caregiving burden, and adaptive processes evolve over time, particularly across key developmental transitions in the child’s life.

There is also a need for more inclusive and diverse samples, with greater representation of fathers and underrepresented populations, in order to capture the full range of parental experiences and reduce gender bias in the literature. In addition, future studies should move beyond descriptive approaches and evaluate the effectiveness of structured psychosocial and family-centred interventions aimed at supporting caregivers. Further research is also needed to explore the impact of diagnostic communication on the parent–child–clinician relationship, with particular attention to how communication practices influence long-term psychological outcomes and engagement with healthcare services. Finally, cross-cultural studies would be valuable to examine how different healthcare systems and sociocultural contexts shape caregiving experiences and access to support.

## Figures and Tables

**Table 1:** Summary of main themes, subthemes, analytical interpretations, and strength of evidence.

Main Theme	Subtheme	Analytical Interpretation (Evidence Pattern)	Type of Evidence	Strength of Evidence	Key Sources
1. Diagnostic journey	1.1 Prolonged uncertainty and diagnostic odyssey	Consistent evidence across studies indicates a prolonged and fragmented diagnostic pathway characterised by multiple consultations, uncertainty, and perceived delays, with convergence between qualitative experiences and quantitative data on diagnostic delay	Qualitative + Quantitative	Strong convergence	[10,28,41]
	1.2 Epistemic injustice and fragmented care	Converging qualitative evidence highlights lack of recognition of parental knowledge and systemic fragmentation, leading parents to assume coordination roles	Qualitative (dominant)	Moderate–strong	[29,31]
	1.3 Moment of diagnosis: shock and validation	Evidence consistently shows diagnosis as an emotional turning point marked by ambivalence (relief + distress) and transition to a new phase of uncertainty	Qualitative + Mixed-methods	Strong	[27,32]
	1.4 Communication as a turning point	Strong convergence indicates that communication quality directly shapes emotional adjustment, trust, and engagement with healthcare services	Qualitative + Mixed-methods	Strong	[27,31]
2. Multidimensional caregiving burden	2.1 Emotional and gendered burden	Robust evidence shows high psychological distress, with consistent gender disparities indicating greater burden among mothers	Quantitative + Mixed-methods	Strong	[35,38]
	2.2 Family reconfiguration	Converging findings indicate substantial reorganisation of family roles, relational strain, and increased caregiving responsibilities	Qualitative + Quantitative	Strong	[26,33]
	2.3 Social isolation and stigma	Consistent qualitative evidence highlights reduced social participation, stigma, and lack of societal awareness	Qualitative + Review	Moderate–strong	[9,18]
	2.4 Economic and physical impact	Evidence indicates significant financial strain, employment reduction, and physical consequences (fatigue, somatic burden), supported by quantitative and survey data	Quantitative + Mixed-methods	Strong	[38,40]
3. Adaptive trajectories	3.1 Coping as a dynamic process	Evidence shows coping as a dynamic and context-dependent process involving both problem-focused and emotion-focused strategies	Quantitative + Qualitative	Strong	[9,37]
	3.2 Parental upskilling and identity transformation	Converging qualitative evidence indicates progressive acquisition of skills and active involvement in care, reflecting role expansion beyond caregiving	Qualitative	Moderate–strong	[18,32]
	3.3 Meaning-making and rebalancing priorities	Evidence highlights ongoing processes of meaning-making, adjustment of expectations, and redefinition of quality of life over time	Qualitative	Moderate	[30,42]

## Data Availability

No new data were created or analyzed in this study. Data sharing is not applicable.

## Supplementary Materials

The following supporting information can be downloaded at https://www.mdpi.com/article/10.3390/healthcare14111437/s1. Table S1, Search strategy; Table S2, QuADS quality appraisal for diverse studies; Table S3, quality appraisal of cross-sectional studies using the JBI Critical Appraisal Checklist; Table S4, Data extraction quantitative studies; Table S5, Data extraction of the mixed-methods studies; Table S6, Data extraction from qualitative studies; Table S7, Data extraction literature reviews.

## Abbreviations

The following abbreviations are used in this manuscript:

JBI	Joanna Briggs Institute
OSF	Open Science Framework
QuADS	Quality Assessment with Diverse Studies
