# Supplementary material for: Integrative Literature Review on the Lived Experiences of Parents of Children with a Rare Disease
Source: Healthcare (Basel). 2026 May 22;14(11):1437. doi: 10.3390/healthcare14111437 (PMC13256770; doi:10.3390/healthcare14111437)
Supplement: Supplementary file 1 [file healthcare-14-01437-s001.zip › healthcare-4305665-supplementary.pdf]

Table S1: Search strategy

Database	Search String	Search results	Included research articles
PubMed	("Rare Diseases"[MeSH Terms] OR "orphan disease"[TIAB] OR "rare disorder"[TIAB] OR "rare disease"[TIAB]) AND ("caregiver"[TIAB] OR "family"[TIAB] OR "carer"[TIAB] OR "parent"[TIAB] OR "mother"[TIAB] OR "father"[TIAB] OR "relative"[TIAB] OR "spouse"[TIAB]) AND ("quality of life"[MeSH Terms] OR "quality of life"[TIAB] OR experiences[TIAB] OR "diagnosis communication"[TIAB] OR "psychological burden"[TIAB] OR "social burden"[TIAB] OR "financial burden"[TIAB] OR "emotional burden"[TIAB])	391	8
CINAHL	("rare disease" OR "rare diseases" OR "rare disorder" OR "rare disorders" OR "orphan disease" OR "orphan diseases" OR "low prevalence disease" OR "low prevalence disorder") AND (caregiver OR caregivers OR carer OR carers OR "family member" OR "family members"OR parent OR parents OR mother OR mothers OR father OR fathers OR spouse OR spouses OR relative OR relatives) AND ("quality of life" OR "psychological burden" OR "social burden" OR "financial burden" OR "emotional burden" OR experiences OR experience OR diagnosis OR "mental burden" OR communication)	691	1
PsycInfo PsycArticle	("rare disease" OR "rare disorder" OR "orphan disease") AND ("caregiver" OR "mother" OR "father" OR "parent") AND ("quality of life" OR "burden" OR "diagnosis communication")	305	1
Scopus	(TITLE-ABS-KEY ("rare disease*" OR "rare disorder*" OR "orphan disease*")) AND (TITLE-ABS-KEY (parent* OR mother* OR father* OR caregiver* OR family)) AND (TITLE-ABS-KEY ("lived experience*" OR burden OR "caregiving burden" OR "quality of life" OR psychosocial OR emotional OR uncertainty OR "diagnostic odyssey" OR "diagnostic delay" OR "diagnostic communication" OR communication)) AND (TITLE-ABS-KEY (child* OR adolescent* OR pediatric* OR paediatric*))	1004	12

Table S2: QuADS Quality Appraisal for Diverse Studies* (adapted from [24])

**QuADS**<br><br>**Quality Appraisal for diverse studies**	**1**<br>Theoretical or conceptual underpinning to the research	**2**<br>Statement of research aim/s	**3**<br>Clear descriptions of research setting and target population	**4**<br>The study design is appropriate to address the stated research aim/s	**5**<br>Appropriate sampling to address the research aim/s	**6**<br>Rationale for choice of data collection tool/s	**7**<br>The format and content of data collection tool is appropriate to address the stated research	**8**<br>Description of data collection procedure	**9**<br>Recruitment data provided	**10**<br>Justification for analytic method selected	**11**<br>The method of analysis was appropriate to answer the research aim/s	**12**<br>Evidence that the research stakeholders have been considered in research design or conduct.	**13**<br>Strengths and limitations critically discussed	**Quality Scores**
**Atkins et al. (2024)** [19]	**2**	**3**	**3**	**3**	**2**	**3**	**2**	**3**	**3**	**2**	**3**	**0**	**3**	**32/39**<br>**Quality= High**
**Bauskis et al. (2022)** [10]	**2**	**3**	**3**	**3**	**2**	**2**	**3**	**3**	**3**	**1**	**3**	**0**	**2**	**30/39**<br>**Quality= High**
**Bayraktar et al. (2024)** [26]	**2**	**3**	**3**	**3**	**2**	**2**	**3**	**3**	**2**	**3**	**3**	**0**	**3**	**32/39**<br>**Quality= High**
**Boettcher et al. (2021)** [36]	**3**	**3**	**2**	**3**	**2**	**3**	**3**	**3**	**3**	**3**	**3**	**0**	**3**	**34/39**<br>**Quality= High**
**Bullock et al. (2026)** [34]	**2**	**3**	**2**	**3**	**2**	**2**	**3**	**3**	**3**	**2**	**3**	**2**	**2**	**32/39**<br>**Quality= High**
**Burbury et al. (2025)** [27]	**3**	**3**	**3**	**3**	**2**	**2**	**3**	**3**	**1**	**3**	**3**	**2**	**3**	**34/39**<br>**Quality= High**
**Carmichael et al. (2015)** [28]	**2**	**3**	**3**	**3**	**3**	**3**	**3**	**3**	**2**	**3**	**3**	**2**	**3**	**36/39**<br>**Quality=High**

**Chu et al. (2022)** [35]	**3**	**3**	**3**	**3**	**2**	**3**	**2**	**2**	**3**	**3**	**2**	**0**	**2**	**31/39 Quality= High**
**Deuitch et al. (2021)** [29]	**2**	**3**	**3**	**3**	**2**	**2**	**2**	**3**	**3**	**2**	**3**	**0**	**2**	**30/39 Quality= High**
**Fitzgerald et al. (2021)** [42]	**2**	**3**	**3**	**3**	**2**	**2**	**3**	**3**	**1**	**2**	**3**	**0**	**2**	**29/39 Quality= Moderate**
**Miller et al. (2023)** [30]	**3**	**3**	**3**	**3**	**2**	**2**	**3**	**3**	**2**	**2**	**3**	**1**	**1**	**31/39 Quality= High**
**Nguyen et al. (2025)** [31]	**2**	**3**	**3**	**3**	**3**	**2**	**3**	**3**	**2**	**2**	**3**	**0**	**2**	**31/39 Quality= High**
**Stafford-Smith et al. (2025)** [32]	**3**	**3**	**3**	**3**	**3**	**3**	**3**	**3**	**3**	**3**	**3**	**1**	**3**	**37/39 Quality= High**
**Von der Lippe et al. (2022)** [18]	**2**	**3**	**3**	**3**	**3**	**2**	**2**	**3**	**3**	**3**	**3**	**0**	**2**	**32/39 Quality= High**
**Witt et al. (2023)** [33]	**1**	**3**	**3**	**3**	**2**	**3**	**3**	**3**	**3**	**3**	**3**	**2**	**2**	**34/39 Quality= High**
**Wu et al. (2024)** [9]	**1**	**3**	**3**	**3**	**3**	**3**	**3**	**3**	**3**	**3**	**3**	**0**	**1**	**32/39 Quality= High**

*Rationale for using QuADS on reviews: QuADS items were adapted to the review context: sampling ↦ search/selection strategy; data collection ↦ data extraction/charting; analysis ↦ narrative synthesis / meta-analysis. This adaptation was pre-specified to ensure consistent appraisal across heterogeneous designs

Table S3: Quality appraisal of cross-sectional studies using the JBI Critical Appraisal Checklist

JBI Criteria	Manalel et al. (2024) [37]	Nazco et al. (2022) [38]	Pavi´c et al. (2024) [39]	Somanadhan et al. (2025) [40]	Walkowiak et al. (2025) [4]	Zurynski et al. (2017) [41]
**1. Were the criteria for inclusion in the sample clearly defined?**	yes	Yes	yes	yes	yes	yes
**2. Were the study subjects and the setting described in detail?**	yes	yes	yes	yes	yes	yes
**3. Was the exposure measured in a valid and reliable way?**	yes	yes	Unclear	yes	yes	yes
**4. Were objective, standard criteria used for measurement of the condition?**	yes	yes	Unclear	Unclear	Unclear	yes
**5. Were confounding factors identified?**	yes	yes	yes	no	no	no
**6. Were strategies to deal with confounding factors stated?**	yes	yes	yes	no	no	no
**7. Were the outcomes measured in a valid and reliable way?**	yes	yes	yes	yes	yes	yes
**8. Was appropriate statistical analysis used?**	yes	yes	yes	yes	yes	yes
**Quality score**	***High 8/8***	***High 8/8***	***Moderate 6/8***	***Moderate 5/8***	***Moderate 5/8***	***Moderate 6/8***

TABLE S4: Data extraction quantitative studies

N	Author (year of publication)	Country	Title	Study type and Aim	Sample and Setting	Data collection tool	Main results	Declared Limitations	Quality Rating
1	Manalel et al. (2024) [37]	USA	Stress, coping, and positive aspects of caregiving among caregivers of children with rare disease.	Cross-sectional observational survey study<br><br>Aim: to investigate whether primary caregiving stress and coping strategies are associated with positive aspects of caregiving (PAC) among caregivers of children with rare diseases, undiagnosed conditions, and typically developing children.	USA<br><br>N = 214 primary caregivers/parents, from 146 families.<br><br>Typically-developing N=62,<br>Rare conditions N=130,<br>Undiagnosed diseases N=22<br><br>Sampling: Non-probability convenience sampling	Instruments:<br><br>Positive Aspects of Caregiving Scale (PAC)<br>The Pediatric ADL of the Children's Habilitation Assessment Tool<br>The Zarit Burden Interview<br>Brief COPE<br><br>Statistical analysis: Generalized estimating equation (GEE) models	•Perceived burden was not associated with PAC.<br>•Venting was negatively associated with PAC ($b= -0.09$, $p=.03$), whereas emotional support was associated with increased PAC for caregivers of children with undiagnosed conditions ($b = 0.15$, $p=.02$).<br>•Care needs were associated with greater PAC among caregivers engaged in high levels of emotional support coping ($b = 0.10$, $p=.01$) and venting ($b = 0.09$, $p=.03$).	-Cross-sectional design, no conclusions about directionality/causality<br>-Measurement limitations<br>-Limited representativeness	Quality Rating= High<br><br>8/8<br>JBI
2	Nazco et al. (2022) [38]	Internatio nal	Health-Related Quality of Life and Perceived Burden of Informal Caregivers of Patients with Rare Diseases in Selected European Countries	Quantitative, cross-sectional, descriptive-correlational study<br><br>Aim: to examine the HRQOL difference between caregivers, and to explore the factors associated with HRQOL	European countries (France, Germany, Italy, Spain, Sweden, UK)<br><br>N = 825 informal caregivers<br>Gender: 80.2% female<br><br>Sampling: non-probability convenience sampling via patient organisations, websites/social media	Instruments:<br><br>Zarit Burden Interview (ZBI)<br>EuroQol-5D (EQ-5D)<br><br>Statistical analysis: Pearson's correlation, ANOVA, multinomial logistic regression	•Caregiver HRQOL (EQ-5D index) was lower with higher burden (ZBI), $r = -0.180$; $p < 0.0001$.<br>• Mobility dimension of EQ-5D was significantly associated with patients age, time devoted to care, patient gender and patient utility index.<br>• Patients' age, burden scores and patient utility index significantly predict the capacity of caregivers to perform activities of daily living.	-Secondary use of data: dataset originally collected for other objectives/analyses<br>-High rate of missing values for caregivers' age	Quality Rating= High<br><br>8/8<br>JBI
3	Pavi´c et al. (2024) [39]	Croatia	The Association between Healthcare Satisfaction and Social Support and Stress, Depression, and Life Satisfaction in Female Caregivers: The Moderating Role of	Cross-sectional study (survey) conducted on a convenience sample<br><br>Aim: To examine the association between healthcare satisfaction and social support with stress, depression, and life satisfaction in female caregivers of children with rare diseases, and to test whether the child's dependence moderates these relationships.	Croatia<br><br>N= 185 female caregivers<br><br>Sampling: non-probability convenience sampling via an emailed online survey link.	Instruments:<br><br>Sociodemographic Data Questionnaire; Child's Dependence Questionnaire; Patient Satisfaction Questionnaire Short Form; Multidimensional Scale of Perceived Social Support; Perceived Stress Scale; CES-D-10; Satisfaction With Life Scale.<br><br>Statistical analysis: Bivariate correlations Hierarchical regression moderation models	•Lower dependence of the child moderated the association between a higher level of healthcare satisfaction and reduced stress and a higher level of life satisfaction.<br>•Lower child dependence moderated the association between a higher level of social support and a reduction in depression. In contrast, this association was absent in female caregivers with highly dependent children.<br>•Higher level of social support led to stress reduction and increased life satisfaction in all respondents.	-Limited number of male respondents<br>-Cross-sectional design (no causal inference)<br>-Self-report measures (social desirability/recall bias)<br>-Cultural and healthcare-system specificity, limited generalizability	Quality Rating= Moderate<br><br>6/8

			Dependence of a Sick Child						
**4**	Somanadhan et al. (2025) [40]	Ireland	Assessing the supportive care needs of parents of children with rare diseases in Ireland	Descriptive quantitative study using an online survey<br><br>Aim:to explore the supportive care needs of parents caring for children with rare diseases in order to inform targeted healthcare services and improve family well-being.	Ireland<br><br>N= 89 (mostly,97,6% parents) 87% female, 13% male<br><br>Sampling: Non-probability convenience sampling	Instruments:<br><br>Parental Needs Scale for Rare Diseases (PNS-RD)<br><br>Statistical analysis: Descriptive statistics; independent-samples t-test	•Parents reported variable unmet supportive care needs across four domains, understanding the disease, working with health professionals, emotional issues, and financial needs.<br>•Emotional strain and financial burden were particularly prominent, including isolation, frustration, difficulties paying for care/equipment, and unmet needs for respite support.<br>•Overall findings highlighted the need for more tailored and coordinated support.	- Low response rate<br>-Limited representativeness of rare diseases<br>-Underrepresentation of fathers<br>-Limited generalizability	Quality Rating= Moderate<br><br>5/8 JBI
**5**	Walkowiak et al. (2024) [4]	Poland	Navigating the Unique Challenges of Caregiving for Children with Rare Diseases: Are the Care Experiences of All Caregivers the Same? A Focus on Life-Limiting Rare Diseases	Cross-sectional comparative study based on a questionnaire survey<br><br>Aim: to compare the differences in the experiences of parenting a child with life-limiting RDs and those with less severe conditions	Poland<br><br>N= 401 caregivers<br>N= 175 caregivers of children with phenylketonuria (PKU) and N= 226 caregivers of children with life-limiting rare diseases (LLRD)<br><br>Sampling: non-probability convenience sampling	Instruments:<br><br>Dd hoc self-administered, anonymous, computer-assisted online questionnaire.<br><br>Statistical analysis: descriptive and comparative analyses; Mann–Whitney U test, chi-square test	•Caregivers of children with LLRD reported a heavier burden than caregivers of children with PKU, including greater emotional distress, personal sacrifice, family conflict, financial strain, and lower ratings of healthcare support.<br>•Findings also indicated important between-group differences in perceptions of the healthcare system and economic challenges	-Limited generalizability<br>-Predominance of mothers/female respondents.<br>-Non-validated questionnaire.	Quality Rating= Moderate<br><br>5/8 JBI
**6**	Zurynski et al. (2017) [41]	Australia	Australian children living with rare diseases: experiences of diagnosis and perceived consequences of diagnostic delays	Cross-sectional observational study<br><br>Aim: to describe the experiences of families of children with rare diseases with regard to diagnosis, diagnostic delays, access to healthcare services, and the impact on the family.	Australia<br><br>N=462 (mother 89%, father 8.5%, both parents 0.9%, (98.4% parents)<br><br>Sampling: non-probability convenience sampling (mail-out to families identified via partner organisations' membership/clinical lists)	Instruments:<br><br>Australian Paediatric Surveillance Unit (APSU) impact on family survey<br><br>Statistical analysis: Frequency distributions + cross-tabulations;<br>median + IQR;<br>thematic coding.	•Diagnostic "odyssey" N=428: 41.8% saw 3–5 doctors; 16.6% saw 6–10; 11.1% saw >10<br>•Perceived delay: 36.7% felt diagnosis could have been earlier; 27.3% received at least one wrong diagnosis first.<br>•Reasons for delay: lack of professional knowledge (69.2%).<br>•Consequences of delay: family stress/frustration/anxiety 54%, disease progression 37%, treatment delay 25%, inappropriate treatments 10%.	-Selection/coverage bias<br>-Response bias	Quality Rating= Moderate<br><br>6/8 JBI

TABLE S5: Data extraction of the mixed-methods studies

N	Author (year of publication)	Country	Title	Study type and Aim	Sample and Setting	Data collection tool	Main results	Declared Limitations	Quality Rating
**1**	Bullock et al. (2026) [34]	Not reported	Parental Experiences of Receiving a Rare Genetic Disease Diagnosis for Their Child on Diagnosis Day	A cross-sectional, mixed-methods survey study, with quantitative descriptive/statistical analyses and inductive thematic analysis<br><br>Aim: to collect insights from parents about the day they received their child's rare genetic disease diagnosis	N= 717 parents (92% mothers, 7% fathers, 1% others)<br><br>Sampling: Non-probability convenience sampling, online recruitment through rare disease organizations' channels and social media.	Instruments:<br>A researcher-developed online survey including closed-ended items and open-ended questions<br><br>Statistical analysis:<br>Descriptive statistics; χ^2 tests (Pearson, Mantel–Haenszel; $p = 0.001$); content analysis and inductive thematic analysis	•Parents described Diagnosis Day as emotionally complex, often with dissatisfaction about how results were communicated.<br>•The most useful information concerned quality of life, clinical management, inheritance, and opportunities to connect with other families, while many parents reported a need for clearer communication, greater empathy, more choice in disclosure, and better support resources.	-Recall bias<br>-Self-reported genetic diagnoses with possible misclassification.<br>-Limited sample diversity<br>-Survey available only in English and Spanish, excluding parents not fluent in either language	Quality Rating = High<br><br>QUADS 32/39
**2**	Chu et al. (2022) [35]	Taiwan	Gender Differences in Caring for Children with Genetic or Rare Diseases: A Mixed-Methods Study	Concurrent triangulation mixed-methods design<br><br>Aim To examine gender differences between male and female caregivers of children with genetic/rare diseases	Taiwan<br><br>N= 100 family caregivers (N= 42 men, N= 58 women)<br><br>Sampling: Non-probability convenience sampling via an outpatient genetic counseling clinic	Instruments:<br>Parenting stress – PIP<br>Depressive symptoms – CES-D short<br>Satisfaction with life scale – SWLS<br><br>Statistical analysis:<br>quantitative group comparisons (male vs female caregivers) using descriptive statistics and inferential tests (t-tests/χ^2), plus qualitative content analysis of the open-ended responses.	•Significant gender differences: female caregivers more daily caregiving hours and tasks; higher parenting stress; more recent depressive symptoms (vs men).<br>•A gender discrepancy in viewpoints about the sequence of three dimensions of cognitive illness perception was found.<br>•Identity may be the key domain of illness perception.	-Limited generalizability<br>-Limited qualitative depth (one brief open-ended item, short responses)	Quality Rating= High<br><br>QUADS 31/39

TABLE S6: Data extraction from qualitative studies

N	Authors (Year of publication)	Country	Title	Aim	Sample and setting	Study design methodology	Analysis	Results	Conclusion, comments, and issue raised	Quality Rating
1	Bauskis et al. (2022) [10]	Australia	The diagnostic odyssey: insights from parents of children living with an undiagnosed condition	To explore the experiences of parents of children living with an undiagnosed condition, particularly in relation to the diagnostic odyssey they faced before receiving a definitive diagnostic outcome	Australia<br>N = 11 families (N = 11 mothers; N = 1 father).	Qualitative study; In-depth semi-structured interviews	Thematic analysis, , verbatim transcription, coding and theme development.	Three main themes:<br>(1) Responding to significant care needs, (2) The diagnostic odyssey, (3) The value of a diagnosis.	The study showed that parents of children living with an undiagnosed condition experienced a substantial emotional and practical burden during the diagnostic odyssey, while also taking on roles as navigators, advocates, and experts in managing their child's complex care needs. A diagnosis was perceived as highly valuable because it could reduce uncertainty, provide explanatory context, improve access to services and supports, and help families plan for the future	Quality Rating= High<br><br>QUADS 30/39
2	Bayraktar et al. (2024) [26]	Turkey	Experiences of mothers caring for children with rare diseases in Turkey	To shed light on the experiences of mothers caring for children with rare diseases	Turkey<br>N= 16 Mothers (mean age 37.18 years)	Descriptive qualitative phenomenological study, semi-structured, face-to-face in-depth interviews	Reflexive thematic analysis; Audio-recorded interviews; verbatim transcription; iterative coding by two researchers; theme development through discussion/consensus	Three main themes:<br>(1) Challenges with Treatment, (2) Burden of Care, (3) Expectations.	Caring for children with rare diseases imposes considerable physical, emotional, and social burdens on mothers.<br>The study reveals significant obstacles in the treatment processes<br>for their children, such as extended diagnosis times, disjointed<br>care due to the absence of a multidisciplinary approach, and the shortage of specialists.	Quality Rating= High<br><br>QUADS 32/39
3	Burbury et al. (2025) [27]	Australia	Exploring the experience of communication in healthcare settings with parents of children with a rare genetic condition: "It's the more negative ones that you remember"	To capture the experiences of communication with genetic and non-genetic healthcare professionals for parents of children with rare conditions (diagnosed and undiagnosed).	Australia<br>N= 14 parents (N=12 mothers and N=2 fathers)	Qualitative study using semi-structured interviews	Reflexive thematic analysis, iterative coding, researcher-driven theme development, and construction of shared meaning through deep data engagement	Six main themes:<br>(1) The need for family-centered Care, (2) The value of à la carte Communication.<br>(3) Technical language is Overwhelming, (4) Negative word choices can be "soul destroying", (5) All results (diagnostic and non-diagnostic) are significant., (6) Where to from here?	The themes highlight the need for personalized, continuous communication using clear and respectful language, as clinical wording can have lasting effects. Parents stressed that all results including non-diagnostic ones carry significant emotional weight, and many felt isolated, underscoring the value of ongoing support from genetic counselors.	Quality Rating= High<br><br>QUADS 34/39

**4**	Carmichael et al. (2015) [28]	USA	"Is it Going to Hurt?": The Impact of the Diagnostic Odyssey on Children and Their Families	To assess the impact of the diagnostic odyssey and to investigate parents' perceptions of the effects of medical procedures on the child, the emotional stress experienced by the family, and the implications associated with obtaining a diagnosis	USA<br><br>N = 10 Mothers	Qualitative study using interpretive description methodology; semi-structured telephone interviews	Audio-recordings with professional transcription, data management, thematic coding using a priori codes from the interview guide alongside emergent themes, and quasi-quantitative analysis of the coded data.	Two main themes:<br>(1) The impact of diagnostic procedures on the child, (2) The value to families of obtaining a diagnosis	This study highlights that obtaining a diagnosis for a child with a rare neuromuscular disorder represents a pivotal moment for families, as it reduces uncertainty, validates parents' concerns, and enables access to support services and future planning. However, during the diagnostic odyssey, families face a substantial emotional and practical burden, often without adequate support.	Quality Rating= High<br><br>QUADS 36/39
**5**	Deuitch et al. (2021) [29]	USA	Doctors can read about it, they can know about it, but they've never lived with it": How parents use social media throughout the diagnostic odyssey	To characterize how parents use social media throughout the diagnostic odyssey (and after diagnosis) to meet informational, social, and emotional support needs.	USA<br><br>N=14 parents (n = 13 mothers, n = 1 father)	Qualitative study using a screening questionnaire + semi-structured interviews	Inductive, team-based coding and thematic analysis based in grounded theory	Four main themes:<br>(1) Parents' struggles to find the "right" community on social media, (2) The value and limits of medical information from social media, (3) The benefits and challenges of finding social support through social media, (4) Balancing privacy and transparency on social media	Social media played a meaningful role across the diagnostic odyssey: parents used it to find community, share and interpret medical information, and gain emotional support, while also facing risks such as misinformation, difficulty finding the "right" group, and ongoing tension between privacy vs. transparency.	Quality Rating= High<br><br>QUADS 30/39
**6**	Fitzgerald et al. (2021) [42]	Ireland	'More than a box of puzzles': Understanding the parental experience of having a child with a rare genetic condition"	To explore parental interpretation, adaption and coping in the context of ambiguous rare genetic findings in order to support parental adjustment and wellbeing.	Ireland<br><br>N= 30 parents (n =24 mothers, n =6 fathers)	Qualitative study using in-depth, semi-structured interviews	Deductive thematic analysis within a constructivist theoretical framework	Three main themes:<br>(1) Learning of the Genetic Diagnosis, (2) The Reality of the Rarity, (3) Beyond Genetics: The Child takes Centre Stage	Findings showed that parents' adjustment to their child's genetic results is shaped by multiple factors, including the child's difficulties and developmental stage, clinician communication, perceptions of genetics, individual coping strategies, access to practical and emotional support, and wider contextual experiences.	Quality Rating= Moderate<br><br>QUADS 29/39
**7**	Miller et al. (2023) [30]	USA	Continuing a search for a diagnosis: the impact of adolescence and family dynamics	To understand how families of children with undiagnosed diseases decide whether to continue pursuing a diagnosis, with attention to the role of adolescence and family dynamics in	USA<br><br>N=14 parents (N= 13 mothers; N=1 Fathers)	Qualitative study; screening survey + semi-structured interviews	Deductive thematic analysis; team-based iterative codebook; consensus coding.	Three main themes:<br>(1) Details of the diagnostic odyssey, (2) Parental duty, (3) Tensions between parent and child	Families often reassess whether to keep pursuing a diagnosis when the diagnostic odyssey becomes prolonged especially during adolescence, when the child's preferences and wellbeing may diverge from parents' drive to "keep searching."	Quality Rating= High<br><br>QUADS 31/39

				that decision-making.						
**8**	Nguyen et al. (2025) [31]	USA	Nobody listened to us for years": Parents' experiences of provider communication in the diagnostic odyssey	To examine parent–clinician communication during the pediatric diagnostic odyssey, and how it may shape families' progress toward a diagnosis and their care experience.	USA<br><br>N=36 parents (N= 33 mothers; N=3 Fathers)	Prospective qualitative study; sequential semi-structured in-depth interviews (3 time points)	Thematic content analysis (inductive + deductive coding)	Three main themes:<br>(1) Provider availability and responsiveness, (2) Trust and validation of parents' concerns, (3) Communication across multiple providers	Parents reported wide variation in communication with providers during the diagnostic odyssey: communication could either facilitate or hinder access to diagnosis and care. Key challenges included poor provider responsiveness, lack of trust and validation of parents' concerns, and fragmented communication across multiple providers.	Quality Rating= High<br><br>QUADS 31/39
**9**	Stafford-Smith et al. (2025) [32]	USA	The book is just being written: The enduring journey of parents of children with emerging-ultrarare disorders	To describe the experiences of parents whose children were diagnosed with an Emerging ultra rare disorder (E-URD) and to explore empowerment in these parents related to the diagnosis	USA<br><br>N= 17 parents (N=13 mothers and N=4 fathers)	Qualitative study using semi-structured interviews	Directed content analysis; initial deductive coding subsequent iterative inductive refinement of transcripts	Parents' responses clustered into three patterns adjusting, managing, and pioneering Empowerment framework:<br>(1) emotional management, (2) connection with similar others, (3) utilization of available resources, (4) partnering with healthcare providers, (5) and seeking new knowledge.	An E-URD diagnosis represents only one stage in an ongoing parental journey. The patterns of adjusting, managing, and pioneering reflect different levels of empowerment and may help guide tailored support, as some parents remain overwhelmed by uncertainty whereas others become increasingly proactive in seeking knowledge, building connections, and advocating for their child and the wider community.	Quality Rating= High<br><br>QUADS 37/39
**10**	Witt et al. (2023) [33]	Germany	Living with a rare disease - experiences and needs in pediatric patients and their parents	To identify the patients' and parents' current experiences in daily life and with the health care system and families' needs and pathways to psychosocial care.	Germany<br><br>N=74 parents (N=63 female, N=11 male) + N=15 pediatric participants.	A multicenter qualitative study, semi-structured telephone interviews	Focused interview analysis using deductive and inductive coding of transcribed interviews.	Five main:<br>(1) daily life with an RD, (2) experiences with the health care system, (3) psychosocial support,<br>(4) difficulties and barriers, and (5) improvements.	Parents reported that a child's rare disease disrupts everyday life, increasing care demands, emotional burden, and often work- and finance-related difficulties. They also described stressful diagnostic and healthcare experiences and a strong need for timely, low-threshold practical and psychosocial support, with support and advocacy groups often experienced as especially helpful.	Quality Rating= High<br><br>QUADS 34/39

TABLE S7: Data extraction literature reviews

N	Authors (Year of publication)	Country	Title	Study Type and Aim	Context and Sample	Data Collection Tool	Main Results	Declared Limitations	QuADS
**1**	Atkins et al. (2024) [19]	International	Living with a Rare Disease: Psychosocial Impacts for Parents and Family Members – a Systematic Review	Systematic literature review<br><br>Aim: to consolidate and summaries published quantitative evidence on the psychosocial impacts experienced by family members of individuals with a rare disease.	N= 30 quantitative studies with N= 5,285 participants, (predominantly mothers) of children with rare diseases.<br><br>Five studies were conducted in the USA and Italy (n=5 each); three in Australia, Germany and China (n=3 each); two in Spain and the UK (n=2 each); and one in Sweden, South Korea, Turkey, Poland and France (n=1 each)	WHOQOL<br>BCFQOL<br>PedsQL-FIM<br>ULQIE<br>Work productivity and activity impairment questionnaire: Specific Health problem (WPAI-SH)<br>Zarit burden inventory	Narrative synthesis<br>Five main domains:<br>(1) emotional and psychological health, (2) quality of life/health-related quality of life, (3) work and occupational, (4) caregiver burden, (5) social and relationships.<br>Key psychosocial impacts included emotional and psychological distress reduced quality of life, work-related difficulties, caregiver burden, and impaired family and social relationships.	- No grey literature / handsearching<br>-Heterogeneity of measures and outcomes<br>-Small samples in primary studies<br>- Limited generalisability across family members: evidence mainly from mothers<br>- Potentially missed eligible studies	Quality Rating= High<br><br>QUADS 32/39
**2**	Boettcher et al. (2021) [36]	International	Being the Pillar for Children with Rare Diseases—A Systematic Review on Parental Quality of Life	Systematic literature review<br><br>Aim: to quantitatively examine the quality of life (QoL) of parents caring for children with rare diseases, providing an overview of shared psychological experiences.	N=31 Quantitative studies cross-sectional designs predominant (28), with a smaller subgroup of cohort studies (3).<br><br>N= 10 countries (Australia, Brazil, Canada, Germany, Iran, Ireland, the Netherlands, Poland, Sweden, United States)	Reported quality-of-life / health-related quality-of-life instruments:<br><br>BCFQOL<br>CarerQol-7D<br>CQOLCF<br>CQOLC<br>PedsQL™ Family Impact Module<br>Short Form Health Survey<br>TAAQoL<br>TYR-QOL<br>ULQIE<br>WHOQOL-BREF-TR	Qualitative Synthesis<br><br>Parents of children with rare diseases generally report a lower quality of life (QoL) than parents of healthy children, particularly in psychological domains. Psychosocial factors, such as stress, anxious and depressive symptoms, social support, and socioeconomic status, appeared to be more important predictors of parental QoL than disease-specific factors.	- Heterogeneity: a wide range of study quality, different QoL instruments, and a limited number of studies per specific rare disease<br>- Limited direct comparisons with other caregiver groups<br>- Weak use of theory<br>-Language restriction, only studies published in English and German	Quality Rating= High<br><br>QUADS 34/39
**3**	von der Lippe et al. (2022) [18]	International	Children with a rare congenital genetic disorder: a systematic review of parent experiences	Systematic literature review<br><br>Aim: to provide an overview of the experiences of parents of children with rare genetic diseases, exploring the associated psychosocial consequences.	N= 33 Qualitative studies<br><br>Parents or primary caregivers of children with variable sample sizes across studies<br><br>N= 15 countries (Canada, UK, USA, Italy, Norway, Australia, Spain, Netherlands, China, Sweden,	The review reports the methodological approach of the included studies, but does not provide a detailed description of the specific data collection instruments used in each study	Thematic synthesis<br>Three main themes:<br>(1) Parents' experiences with health care<br>(2) Responsibilities and challenges and (3) Factors promoting positive experiences in parents	- Challenges extracting/synthesizing data from the included qualitative papers<br>- Search restrictions may have led to missing relevant studies	Quality Rating= High<br><br><br>QUADS 32/39

					Denmark, Ireland, Taiwan, New Zealand, Montenegro).				
**4**	Wu et al. (2024) [9]	International	Caregiving experiences of caregivers of children with rare diseases: A qualitative meta-synthesis	A qualitative meta-synthesis<br><br>Aim: to synthesise findings from qualitative research on the experiences and unmet needs of caregivers of children with rare diseases.	N=20 qualitative studies with N= 348 caregivers (N= 79 fathers, N= 232 mothers, N= 1 grandmother)<br><br>Countries represented: Canada; Spain, Italy, United States, United Kingdom, Australia, China; Netherlands, Sweden, Norway, Turkey	The review identifies the broad data collection approaches adopted in the included studies, but does not detail the specific instruments used in each one	Thematic synthesis<br>Three main themes:<br>(1) Life is changed by rare<br>(2) Many unmet needs<br>(3) Strive to adapt and grow	- Language/selection bias<br>- Cultural differences affecting interpretation<br>- Reporting bias	Quality Rating= High<br><br>QUADS 32/39
